# Deterioration of Cough, Respiratory, and Vocal Cord Functions in Patients with Multiple System Atrophy

**DOI:** 10.3390/neurolint15040077

**Published:** 2023-10-02

**Authors:** Takashi Asakawa, Mieko Ogino, Naomi Tominaga, Naoto Ozaki, Jin Kubo, Wataru Kakuda

**Affiliations:** 1 Department of Clinical Medical Sciences, Rehabilitation Medicine, Graduate School of Medicine, International University of Health and Welfare, Narita 286-8686, Japan; takashi.rpt@gmail.com; 2 Department of Rehabilitation, Division of Physiotherapy, International University of Health and Welfare, Ichikawa Hospital, Ichikawa 272-0827, Japan; 3 Department of Neurology, Intractable Neurological Disease Center, International University of Health and Welfare, Ichikawa Hospital, Ichikawa 272-0827, Japan; ogino@iuhw.ac.jp (M.O.); natominaga@iuhw.ac.jp (N.T.); 4 Department of Rehabilitation Medicine, School of Medicine, The Jikei University, Minato-ku 105-8471, Japan; nozakiame@chibamed.org; 5 Department of Rehabilitation Medicine, School of Medicine, International University of Health and Welfare, Narita 286-8686, Japan; j.kubo@iuhw.ac.jp

**Keywords:** multiple system atrophy (MSA), peak cough flow (PCF), cough function, respiratory function, neurodegenerative disease

## Abstract

The purpose of this study was to clarify changes in cough function in patients with multiple system atrophy (MSA). Seventeen probable patients with MSA were studied. Peak cough flow (PCF), respiratory function (percentage of vital capacity, percentage of forced vital capacity, and percentage of predicted forced expiratory volume in one second), respiratory muscle strength (percentage of maximal inspiratory mouth pressure and percentage of maximal expiratory mouth pressure), and maximum phonation time (MPT) were assessed. Walking ability, disease duration, possibility of air stacking, Unified MSA Rating Scale (UMSARS), and Movement Disorder Society Unified Parkinson’s Disease Rating Scale (MDS-UPDRS) Part III were also assessed. Data were separately analyzed for ambulatory and non-ambulatory groups categorized by Functional Ambulation Categories. PCF, respiratory function, respiratory muscle strength, and MPT were significantly lower in the non-ambulatory group than in the ambulatory group. On the other hand, no correlation between PCF and disease duration was observed. A significant number of patients in the non-ambulatory group were unable to hold their breath. The UMSARS and MDS-UPDRS Part III in the non-ambulatory group were significantly higher than in the ambulatory group. It was concluded that ambulatory dysfunction is associated with the decline of cough function and respiratory-related function in patients with MSA.

## 1. Introduction

Multiple system atrophy (MSA) is a rare and progressive neurodegenerative disorder, including olivopontocerebellar atrophy (OPCA), Shy–Drager syndrome (SDS), and striatonigral degeneration (SND) [1,2]. MSA is classified into MSA with predominant cerebellar ataxia (MSA-C) based on OPCA, as cerebellar symptoms are the main symptoms, and MSA with predominant parkinsonian features (MSA-P) based on SND, as parkinsonism is the main symptom [3]. SDS, whose main symptoms are autonomic dysfunction, is not used as a disease type classification. The average life expectancy of patients with MSA is reported to be 9.8 years [4,5]. During disease progression, aspiration pneumonia becomes an important factor that decreases the activities of daily living (ADL) and quality of life (QOL) and is one of the causes of mortality in patients with MSA [6,7]. In addition, the causes of aspiration pneumonia are multifactorial, and a decreased cough function is reported as one of them [8]. In clinical practice, gastrostomy tube feeding is considered for patients with an insufficient cough function, and a tracheostomy is performed for the management of upper airway clearance in patients with MSA [1,9,10]. Therefore, it is important to understand cough function in patients with MSA. Symptomatic treatment is the main treatment for various symptoms, since no fundamental treatment for patients with MSA has yet been established [3]. Some researchers conclude that respiratory function is maintained in spite of disease progression in patients with MSA, while others conclude that it deteriorates [11,12]. In addition, vocal cord dysfunction is strongly associated with life-threatening events such as nocturnal sleep apnea syndrome and stridor in patients with MSA. Respiratory function and vocal cord function play an important rule to produce cough movement [13]. Therefore, when evaluating cough function in patients with MSA, it is important to evaluate not only respiratory function but also vocal cord function. Regarding cough assessment, two points for the peak cough flow (PCF) value, which are 270 L/min and 160 L/min, are used as criteria for evaluating cough strength. When PCF is less than 270 L/min, it becomes difficult to produce sufficient sputum, so other methods of sputum excretion are required [14,15]. When PCF falls below 160 L/min, suction or endotracheal intubation is required [15,16]. These two points are important when assessing cough function. However, it is unclear how these functions change in patients with disease progression. In particular, no researcher has investigated the association between the deterioration of walking capability and the development of cough dysfunction in patients with MSA so far. Clarifying this issue would be clinically helpful for planning therapeutic interventions for patients with MSA. Furthermore, it would be useful when conducting rehabilitation interventions aimed at treating aspiration pneumonia in patients with MSA. Therefore, the purposes of this study were to (1) clarify changes in cough function due to disease progression and (2) reveal the change in other respiratory-related functions that influence cough function.

## 2. Materials and Methods

### 2.1. Ethical Considerations

This study was approved by Medical Ethics Committee of International University of Health and Welfare School of Medicine (approval number: 21-Im-006) and registered with University Hospital Medical Information Network (UMIN) center (UMIN-CTR ID: R000051816 UMIN000045378). This study was conducted in accordance with the Declaration of Helsinki, and written informed consent was obtained from all participants before starting the research.

### 2.2. Participants

This study included Japanese patients who were diagnosed with probable MSA, based on the second consensus diagnostic criteria [17]. All participants were admitted to International University of Health and Welfare Ichikawa Hospital Intractable Disease Center (Chiba, Japan) between July 2021 and December 2022. Participants in this study were recruited from those admitted to the Department of Neurology of our hospital. In addition, we requested that the MSA registry, which has accumulated information on patients with MSA and cooperates in research, post our research offline and recruit participants.

The inclusion criteria for this study were as follows: (1) clinically diagnosed with probable MSA according to the second consensus diagnostic criteria [17]; (2) no respiratory disease such as chronic obstructive pulmonary disease, no heart disease such as myocardial infarction or cardiac insufficiency, no malignant tumors, no other neurological or neuromuscular disease other than MSA, and no acute or chronic inflammatory or infectious diseases; (3) no tracheostomy and/or tracheostomy ventilation therapy; (4) able to verbally communicate; and (5) no cognitive decline (based on Revised Hasegawa’s Dementia Scale (HDS-R) score < 20, Mini-Mental State Examination (MMSE) score < 21, and Frontal Assessment Battery (FAB) score < 13); (6) able to understand the purpose and methods of this study and willing to enter this investigation as a study participant.

### 2.3. Outcome Measure

PCF represents the maximum expiratory flow during coughing and is one of the simple cough function evaluations [14]. Therefore, PCF was selected as the primary outcome measure. All participants were seated in a reclining position with a face mask (PHILIPS, cough assist face mask) placed over the mouth and nose, with careful attention paid by the examiner to ensure a fit that prevents air leaks. A face mask was used instead of a mouthpiece for PCF measurement because of the possibility of leakage due to opening and closing mouth disorders. The face mask was connected to a peak flow meter (Clement Clarke International, Mini-Wright Standard Peak Flow Meter, Essex, UK) to assess peak cough flow. Participants were instructed to cough after maximal deep inspiration. PCF assessment was performed 3 times, and the highest value was used for analysis [18].

Spirometry (Minato medical science Co., Ltd., Autospiro AS-407, Osaka, Japan) was used for respiratory function assessment. The percentage of vital capacity (%VC), percentage of forced vital capacity (%FVC), and percentage of predicted forced expiratory volume in one second (%FEV1.0), corrected for sex, age, height, and weight, were measured by spirometry. As with the PCF measurement, a face mask was coupled to a spirometer, and the examiner covered the nose and mouth to prevent air leaks. Respiratory muscle strength was measured using a respiratory muscle strength measuring device (KOBATA GAUGEg MFG. Co., Ltd., IOP-01, Osaka, Japan) conforming to the recommended American Thoracic Society and European Respiratory Society methods of oral manometry [19,20]. The percentage of maximal inspiratory mouth pressure (%PImax) and percentage of maximal expiratory mouth pressure (%PEmax), corrected for sex, age, height, and weight, were measured by the device. These values were obtained by wearing a nose plug and firmly holding the mouthpiece connected with the device and mouth with fingers to prevent air leaks. %PImax was measured after participants were asked to inspire with as much force as possible. %PEmax was, conversely, measured from the starting point of full inspiration (total lung capacity) to the completion of exhaling with as much force as possible [21]. Both respiratory function assessment and respiratory muscle strength testing were performed after practicing once. For MPT assessment, the participants pronounced the vowel /a/ for as long as possible after single maximal inspiration, and we measured the time [22]. The feasibility of air stacking was determined by checking for air leakage due to exhalation after deep inspiration. Specifically, a face mask with a simple spirometer (Haloscale standard respirometer, nSpire Health, Ltd., London, UK) was placed in close contact with each participant’s face, with instructions to hold their breath after taking a deep inspiration. If the expiratory flow was not measured by the simple spirometer, it was determined that air stacking was possible, and, in cases where expiratory flow was observed, it was determined that air stacking was impossible.

All participants were assessed regarding severity by Unified Multiple System Atrophy Rating Scale (UMSARS) Parts I, II, and IV and the Hoehn and Yahr Scale (H&Y) and motor severity using the Movement Disorder Society Unified Parkinson’s Disease Rating Scale (MDS-UPDRS) Part III. The UMSARS Part I evaluates impairments in activities of daily living, Part II evaluates motor dysfunctions, and Part IV evaluates the global disability level. Parts I, II, and IV consist of questions (12, 14, and 1, respectively), and each part of items are scored from 0 (unaffected) to 4 (severe impairment) [23]. H&Y is widely used to classify the severity of Parkinson’s symptoms. It has 5 stages of evaluations, with stage 1 consisting of unilateral involvement and stage 5 consisting of being wheelchair-bound or bedridden [24]. MDS-UPDRS Part III has 33 assessment items scored from 0 (normal) to 4 (severe) to clarify the severity of motor function for parkinsonism [25]. Scale for the Assessment and Rating of Ataxia (SARA) was used to assess ataxia. The SARA consists of 8 items, with a score from 0 (non-ataxia) to 40 (the most severe ataxia) points. The 8 assessment items are as follows: walking, standing, sitting, speech impairment, finger chasing test, finger–nose test, hand pronation/supination movement, and heel–shin test [26]. Cognitive function was assessed using MMSE and FAB. As basic information on the participants, sex (F/M), height (cm), weight (kg), BMI (kg/m^2^), age at onset (years), disease duration (months), and disease sub-type were collected from medical records. For this study, participants were divided into two groups based on Functional Ambulatory Categories (FACs) [27]. According to previous research, inability to independently walk was rated as 0–2 in FACs [28]. Therefore, participants were divided into an “ambulatory group” (FACs: 5, 4, and 3) and a “non-ambulatory group” (FACs: 2, 1, and 0) in this study. All measurements, examinations, and assessments were performed by trained physiotherapists, speech and language therapists, neurologists, and rehabilitation physicians.

### 2.4. Statistical Analysis

Statistical analyses were performed using software (IBM Corp., Armonk, SPSS statistics version 27). Data are expressed as the median and interquartile ranges for non-parametric variables and ordinal data. The numbers and percentages are reported for nominal data and the ratio scale. The Mann–Whitney U test was used to compare non-parametric variables between ambulatory and non-ambulatory groups. Fisher’s exact test was employed to analyze categorical data between the 2 groups. Spearman’s rank correlation coefficient was used to examine the relationship between the 2 variables. A probability value of <0.05 was defined as significant.

## 3. Results

### 3.1. Demographic and Clinical Characteristics

We enrolled 22 consecutive patients with probable MSA during the research period. Of these 22 patients, 5 did not meet the inclusion criteria (3 were on a tracheostomy ventilator, 1 had cognitive decline, and 1 did not wish to participate in this study). Therefore, 17 consecutive patients with MSA were examined for this study.

The participants’ backgrounds are shown in Table 1. Of the 17 patients, 11 were able to independently walk using a walker or cane (ambulatory group). The remaining six were unable to walk on their own (non-ambulatory group). No significant difference between the two groups for any item of basic information or cognitive function was observed. In contrast, a significant difference in motor function was observed between the two groups. Importantly, the weight of the non-ambulatory group was less than 50 kg. This is because the two groups had a non-normal distribution, and the values were shown as medians.

### 3.2. PCF and Other Respiratory-Related Function

**Figure 1:**
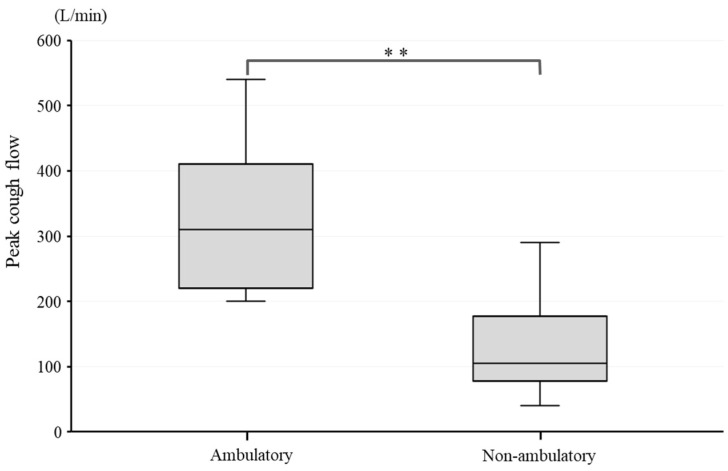
PCF difference between ambulatory and non-ambulatory groups. **: *p* < 0.01.

**Figure 2:**
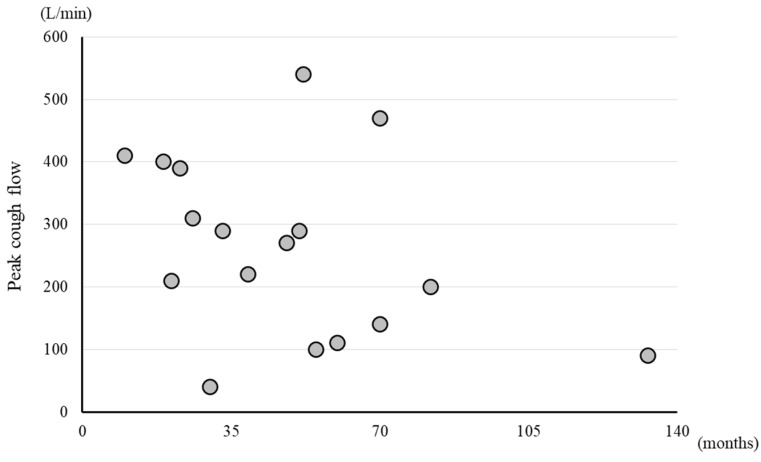
Scatter plots of PCF and disease duration. Circle symbol: PCF value of each participant.

PCF was significantly higher in the ambulatory group than in the non-ambulatory group (*p* < 0.01). The median PCF was 310 L/min (245–405) in the ambulatory group and 105 L/min (92.5–132.5) in the non-ambulatory group (Figure 1). A weak negative correlation was observed between PCF and disease duration, with a correlation coefficient, r, of −0.398, although the correlation was statistically non-significant (*p* = 0.114) (Figure 2).

Table 2 shows the difference in respiratory muscle strength and respiratory function between the two groups. %PImax and %PEmax were significantly higher in the ambulatory group than in the non-ambulatory group (*p* < 0.01). In addition, %VC, %FVC, and %FEV_1.0_ were also significantly higher in the ambulatory group than in the non-ambulatory group (*p* < 0.01).

Table 3 shows the difference in vocal cord function between the two groups. The results showed a significant correlation between the ability to hold breath (air stacking possibility) and walking ability (*p* < 0.01). A difference in frequency due to the adjusted residuals was found, and the association coefficient, which indicates the degree of association, was also significant (φ = −0.849). In addition, MPT was significantly longer in the ambulatory group compared to the non-ambulatory group (*p* < 0.05).

Table 4 shows a summary of the abdominal function of the two groups in the UMSARS Part 2 and MDS-UPDRS Part 3 evaluations. The UMSARS Parts 2–6 score (degree of rigidity) was significantly higher in the non-ambulatory group than in the ambulatory group (*p* < 0.05). Also, the total score of UMSARS Parts 2–11, 12, 13, and 14 (degree of trunk ataxia) was significantly higher in the non-ambulatory group than in the ambulatory group (*p* < 0.01). Similarly, the MDS-UPDRS Parts 3–3 score (degree of rigidity) was significantly higher in the non-ambulatory group than in the ambulatory group (*p* < 0.01). The total score of MDS-UPDRS Parts 3–9, 10, 12, and 13 (degree of trunk motor function) was significantly higher in the non-ambulatory than in the ambulatory group (*p* < 0.01).

## 4. Discussion

This study showed that the decline of walking function is significantly associated with the deterioration of coughing and respiratory function in patients with MSA. On the other hand, no correlation was observed between the severity of cough dysfunction and disease duration in such patients.

Walking ability based on ADL in patients with MSA was focused on this study. From the perspective of ADL milestones in patients with MSA, it was already reported that the median times from onset to aid-required walking and to the need for a wheelchair were 3 and 5 years, respectively [29]. The participants in the present study were a group showing a similar disease duration and ADL deterioration as described in natural history reports. Previous studies on cough strength showed that when the PCF value is 270 L/min or less and respiratory infections develop, acute respiratory failure may occur. Endotracheal intubation should be considered when PCF is 160 L/min or less [15]. In addition, 31–78% of patients with MSA report dysphagia within 5 years [9]. From the results of this study, it was found that sufficient coughing ability was lost during the non-ambulatory period, and patients in the non-ambulatory group were at a high risk of aspiration pneumonia. Therefore, it can be suggested that (1) ADL evaluation, especially the assessment of walking ability, is important to speculate cough ability; (2) attention should be paid to the possibility that cough strength may decrease when walking ability declines; and (3) cough function does not depend on disease duration, which means regular cough evaluation is necessary even if the patient is ambulatory.

Coughing mainly comprises three factors: (1) the acquisition of deep inspiration before coughing, (2) the closure of vocal cords, and (3) an increase in intrathoracic pressure due to the contraction of trunk muscles [30]. First, regarding the pre-cough deep inspiration, some previous studies concluded that respiratory function in patients with MSA is gradually impaired, while others showed that it is not [11,12]. In this study, %VC, %FVC, and %FEV1.0 were lower in the non-ambulatory group than in the ambulatory group. In addition, the reduction in %PImax and %PEmax was marked in the non-ambulatory group compared with the ambulatory group. From these findings, one of the reasons for the decrease in cough function might be that respiratory muscle weakness could occur when walking ability declines in patients with MSA and that decreased respiratory muscle strength makes it difficult to self-acquire deep inspiration.

The ability to close the vocal cords without air leakage plays an important role in amplifying intrathoracic pressure [30]. In this study, the non-ambulatory group performed insufficient air stacking after deep inspiration. MPT is used as an indirect assessment of laryngeal and vocal cord movements, and its relationship with aspiration is known [31]. In addition, it was reported that MPT can be an indicator of the glottic closure required for intrathoracic compression in the process of coughing [32,33]. It is straightforward to understand that MPT depends on the vital capacity, age, and sex. Furthermore, the cut-off value of MPT in patients with MSA has not been defined. However, in the non-ambulatory group, the inability to perform air stacking and the decrease in MPT indicate deterioration in vocal cord motor function and insufficient intrathoracic compression. Therefore, it is suggested that decreased vocal cord function in patients with MSA leads to an insufficient reduction in intrathoracic pressure and decreased PCF.

After extensive research, no studies on abdominal muscle functions and coughing in patients with MSA were identified. On the other hand, the activity of abdominal muscles during coughing in patients with Parkinson’s disease (PD) was found to be decreased, regarding the maximum amplitude of abdominal electromyographic (EMG) activity and the delay in the rate of a rise in activities [34]. Many studies already reported that rigidity and bradykinesia are related to abdominal muscle contractions that coordinate with inspiratory muscle activity during deep inspiration, adduction, abduction of the vocal cords during compression time, and abdominal expiratory muscle activity during expiration time in patients with PD [35,36]. It was attempted to evaluate abdominal function by the clinically convenient and commonly used UMSARS Part 2 and UPDRS Part 3 total scores and their selected sub-items’ score. As a result, the non-ambulatory group showed more severe impairments than the ambulatory group in terms of rigidity, abdominal ataxia, and abdominal motor function. In addition, as described above, %PEmax significantly decreased in the non-ambulatory group. It can be speculated that the result was not due to expiratory muscle weakness but a muscle output weakness resulting from rigidity and ataxia. Therefore, the result of this study suggests that patients with MSA showing impaired walking ability may present with severe rigidity and ataxia including asynergia, adiadochokinesia, and dyschronometria. These neurological symptoms may cause the decrease of coordinated muscle activities and intrathoracic pressure, leading to the insufficiency of producing an effective cough.

This study had some limitations. First, the sample size was relatively small. In total, 17 patients participated in this clinical study. According to an epidemiological survey, approximately 12,000 patients with MSA are in Japan. MSA is one of the rarest diseases not only in Japan but also in other countries. The sample size for previous MSA research was around 20–50 participants. Although the sample size of this study was small, the findings of this study do not have a small clinical significance for improving the quality of interventions for patients with MSA. To increase research precision, it is necessary to conduct research with a larger number of participants. Second, this study was a single-center intervention. Single-center studies tend to be associated with a higher risk of bias and are more prone to the overestimation of intervention effects than multi-center studies. Third, this was a cross-sectional study. As a future direction, a longitudinal evaluation by following the same patients over time is necessary to examine the chronological changes in cough function in patients with MSA. In addition, it may be meaningful to investigate whether a rehabilitative intervention could influence the deterioration of cough and respiratory function in patients with MSA.

## 5. Conclusions

In patients with MSA, ambulatory dysfunction was associated with the reduction in cough and respiratory function. In such patients, furthermore, it was shown that trunk and vocal cord function gradually deteriorated with disease progression. A possible application of the results of this study is that it may be possible to predict deterioration in cough function and respiratory function by simply assessing walking function. Therefore, it is recommended to periodically evaluate walking function in patients with MSA.

## Figures and Tables

**Table 1:** Participants’ information.

	All (N = 17)	Ambulatory Group (N = 11)	Non-Ambulatory Group (N = 6)	*p*-Value
Basic information				
Gender (F/M)	8/9	4/7	4/2	0.247
Height (cm)	162.0 (157–171)	168.0 (159.5–171.5)	159.0 (157.3–161.5)	0.256
Weight (kg)	58.0 (43.8–62)	59.0 (52.7–67)	42.8 (40.4–57.5)	0.180
BMI (kg/m^2^)	20.3 (18–24.8)	21.0 (20.2–24.9)	18.1 (16.5–20.7)	0.216
Age at onset (years)	55.5 (53–63.9)	53.9 (51.2–64.5)	56.5 (53.5–60)	0.733
Disease duration (months)	48.0 (26–60)	39.0 (22–51.5)	57.5 (38.5–67.5)	0.122
Sub-types (MSA-P/MSA-C)	6/11	3/8	3/3	0.339
Cognitive function				
HDS-R	28.0 (26–29)	28.0 (25.5–29)	28.5 (27.3–29)	0.462
MMSE (points)	28.0 (26.5–29)	28.0 (26.3–28.8)	28.0 (27–30)	0.768
FAB (points)	14 (14–16.5)	15 (14–16)	14 (14–18)	0.679
Motor function				
FAC (N)	17	11	6	
5	-	4	-	-
4	-	7	-	-
3	-	0	-	-
2	-	-	0	-
1	-	-	3	-
0	-	-	3	-
Hoehn and Yahr (total)	4 (3–5)	3 (3–4)	5 (5–5)	<0.01
I	0	0	0	-
II	1	1	0	-
III	5	5	0	-
IV	4	3	1	-
V	7	2	5	-
SARA (points)	17.5 (15.5–25)	16.0 (15–18.5)	28.3 (25.5–32.1)	<0.01
MDS-UPDRS Part 3 (points)	41.0 (34–67)	36.0 (29.5–45)	71.5 (68–79.5)	<0.01
UMSARS Part 1 (points)	22.0 (20–29)	20.0 (19–21.5)	35.5 (30.5–38.3)	<0.01
UMSARS Part 2 (points)	20.0 (16–33)	16.0 (15.5–21)	35.5 (33.5–37.5)	<0.01
UMSARS Part 4 (points)	2 (2–4)	2 (2–2)	4 (4–4.8)	<0.01

Abbreviations: BMI: body mass index; MAS-P: MSA with predominant parkinsonian features; MSA-C: MSA with predominant cerebellar ataxia; HDS-R: Revised Hasegawa’s Dementia Scale; MMSE: Mini-Mental State Examination; FAB: Frontal Assessment Battery; FAC: Functional Ambulatory Categories; SARA: Scale for the Assessment and Rating of Ataxia; MDS-UPDRS: Movement Disorder Society Unified Parkinson’s Disease Rating Scale; UMSARS: Unified Multiple System Atrophy Rating Scale.

**Table 2:** Results of respiratory muscle strength and function in both groups.

		Ambulatory Group	Non-Ambulatory Group	*p*-Value
Respiratory2735muscle strength	%PImax (%)	65.8 (54.6–92.7)	18.4 (14.4–30.4)	<0.01
	%PEmax (%)	63.3 (53.9–71.8)	25.8 (21.5–33.7)	<0.01
Respiratory function	%VC (%)	84.0 (74.0–92.5)	55.5 (38.8–62.5)	<0.01
	%FVC (%)	84.0 (74.5–93.5)	47.0 (35.5–52.5)	<0.01
	%FEV_1.0_ (%)	89.0 (81.0–92.0)	45.5 (33.3–51.8)	<0.01

Abbreviations: %PImax: percentage of maximum inspiratory mouth pressure; %PEmax: percentage of maximum expiratory mouth pressure; %VC: percentage of vital capacity; %FVC: percentage of forced vital capacity; %FEV_1.0_: percentage of predicted forced expiratory volume in one second.

**Table 3:** Results of air stacking possibility and MPT as evaluations of vocal cord function.

	Ambulatory Group	Non-Ambulatory Group	*p*-Value	φ
Air stacking possible	11	1	<0.01	−0.874
Air stacking impossible	0	5		
MPT (sec)	20.0 (17.3–21.0)	13.1 (10.2–17.3)	<0.05	-

Abbreviation: MPT: maximum phonation time.

**Table 4:** Sub-item results of UMSARS and MDS-UPDRS as assessments of abdominal motor function.

	Ambulatory Group	Non-Ambulatory Group	*p*-Value
Assessment Scale			
UMSARS Parts 2–6	0 (0–1.5)	2 (2–2)	<0.05
UMSARS Parts 2–11, 12, 13, and 14	7 (5–8.5)	13 (12.3–13.8)	<0.01
MDS-UPDRS Parts 3–3	0 (0–4)	12.5 (10.5–14.5)	<0.01
MDS-UPDRS Parts 3–9, 10, 12, and 13	7 (5.5–9.5)	15 (15–15)	<0.01

Abbreviations: UMSARS: Unified Multiple System Atrophy Rating Scale; MDS-UPDRS: Movement Disorder Society-sponsored revision of the Unified Parkinson’s Disease Rating Scale.

## Data Availability

The data that support the findings of this study are available from the corresponding author upon reasonable request.
